# Roles of MicroRNAs in Bone Destruction of Rheumatoid Arthritis

**DOI:** 10.3389/fcell.2020.600867

**Published:** 2020-11-19

**Authors:** Hanxiao Zhao, Aiping Lu, Xiaojuan He

**Affiliations:** ^1^Institute of Basic Research in Clinical Medicine, China Academy of Chinese Medical Sciences, Beijing, China; ^2^The Second Clinical College of Guangzhou University of Chinese Medicine, Guangzhou, China; ^3^Law Sau Fai Institute for Advancing Translational Medicine in Bone and Joint Diseases, School of Chinese Medicine, Hong Kong Baptist University, Kowloon Tong, Hong Kong; ^4^Shanghai GuangHua Hospital of Integrated Traditional Chinese and Western Medicine, Institute of Arthritis Research, Shanghai Academy of Chinese Medical Sciences, Shanghai, China

**Keywords:** microRNAs, rheumatoid arthritis, bone destruction, osteoclast, osteoblast

## Abstract

As an important pathological result of rheumatoid arthritis (RA), bone destruction will lead to joint injury and dysfunction. The imbalance of bone metabolism caused by increased osteoclast activities and decreased osteoblast activities is the main cause of bone destruction in RA. MicroRNAs (MiRNAs) play an important role in regulating bone metabolic network. Recent studies have shown that miRNAs play indispensable roles in the occurrence and development of bone-related diseases including RA. In this paper, the role of miRNAs in regulating bone destruction of RA in recent years, especially the differentiation and activities of osteoclast and osteoblast, is reviewed. Our results will not only help provide ideas for further studies on miRNAs’ roles in regulating bone destruction, but give candidate targets for miRNAs-based drugs research in bone destruction therapy of RA as well.

## Introduction

Rheumatoid arthritis (RA) is the most common systemic inflammatory arthritis. As one of the major public health challenges in the world, the standardized prevalence rate of RA is 240 per 100,000 population, and its prevalence and morbidity are currently on the rise (Safiri et al., 2019). Bone destruction is a hallmark of this disease and occurs in the following forms: focal bone at the edge of the joint and subchondral erosion, bone loss around joints and systemic bone loss (osteoporosis) (Goldring, 2015). It occurs in the early stage of RA and develops with time, leading to joint damage and functional impairment in patients, which is an important prognostic indicator in RA (Panagopoulos and Lambrou, 2018). Bone homeostasis relies on the balance between osteoclasts-mediated bone absorption and osteoblasts-mediated bone formation (Guo Q. et al., 2018). Bone destruction in RA is an imbalance of this relationship, which is the result of inducing osteoclasts and inhibiting osteoblasts (Panagopoulos and Lambrou, 2018).

MicroRNAs (MiRNAs) are an endogenous, small (about 22 nucleotides long), non-coding and functional RNA families. MiRNAs are differentially expressed and deregulated in RA, and participate in the pathogenesis of RA by regulating target genes (Li et al., 2019). The association between changes in miRNAs expression and pathogenesis of RA has been previously demonstrated (Sugatani et al., 2011). Because miRNAs have multiple functions and extensive regulatory functions, they also play important roles in bone pathophysiology. MiRNAs have become an important regulator of osteoclastogenesis, osteoblasts growth and differentiation (Chen et al., 2015; Baum and Gravallese, 2016; Liu J. et al., 2019). In experimental arthritis models, miRNAs analogs and antagonists have shown encouraging results as experimental treatments (Hou et al., 2019).

Therefore, the purpose of this article is to comprehensively review recently reported miRNAs in osteoclastogenesis and osteoblastogenesis, highlighting their pathogenetic mechanisms leading to the development of bone destruction in RA. We searched Medline database through PubMed for English language original articles from 2016 to 2020 using keywords as the following: rheumatoid arthritis and miRNAs and bone destruction, rheumatoid arthritis and osteoclast and miRNAs, rheumatoid arthritis and osteoblast and miRNAs, rheumatoid arthritis and miRNAs and bone, osteoclast differentiation and miRNAs, osteoblast differentiation and miRNAs, osteoclastogenesis and miRNAs, and osteoblastogenesis and miRNAs. The miRNA-target interaction proposed by bioinformatics alone were not be discussed here. Reviews and articles in other languages were excluded. Bone damage caused by other factors and diseases, changes in miRNAs caused by drugs or other factors, as well as indirect effects of miRNAs on osteoclasts and osteoblasts on bone damage were excluded.

## Bone Destruction in RA

The cores of bone destruction in RA lie in excessive bone absorption and insufficient bone reconstruction. Osteoclasts play a key role in the bone destruction of RA. Monocytes of the monocyte/macrophage lineage are precursors of osteoclasts, which enter the inflamed joints and synovium and differentiate into osteoclasts (Karmakar et al., 2010). Osteoclasts differentiation can be regulated through receptor activator for nuclear factor-κ B ligand (RANKL), which binds to its receptor activator for nuclear factor-κB (RANK), and macrophage colony-stimulating factor (M-CSF), which binds to its receptor colony stimulating factor 1 receptor (CSF1R, also known as M-CSFR) (Lozano et al., 2019). Osteoblasts, synovial fibroblasts (FLS) and other cells express RANKL, and RANKL binds with its receptor RANK, which activates osteoclasts to generate signals (Ono et al., 2020). Driven by RANKL receptor activator, the activation of nuclear factor of activated T cells (NFATc1) and tumor necrosis factor receptor-related factor 6 (TRAF6) can further promote the activation and expression of other transcription factors, signal pathways and genes required for osteoclast differentiation (Chen X. et al., 2018; Hrdlicka et al., 2019). Leucine rich repeat containing G protein-coupled receptor 4 (LGR4) is another receptor of RANKL, which competes with RANK to bind RANKL and inhibits the typical RANKL signal during osteoclast differentiation (Luo et al., 2016). Osteoprotegerin (OPG) is a receptor for RANKL and competitively inhibits RANK/RANKL binding. In RA, the ratio of RANKL and OPG can affect this disease progression (Panagopoulos and Lambrou, 2018). M-CSFR, the receptor encoded by c-fms gene, is dimerized, phosphorylated, and activated by M-CSF, which triggers the activation of serine/threonine kinase family called mitogen-activated protein kinase (MAPK) and phosphatidylinositol 3 kinase (PI3K). The activation of PI3K stimulates the activation of protein kinase B (PKB, or AKT) and mammalian target of rapamycin (mTOR). This process plays a role of proliferation and survival in osteogenesis (Anesi et al., 2019).

The microenvironment of RA can not only promote the production and activation of osteoclasts, but also inhibit the function of the osteoblasts (Gravallese, 2017). Due to the limited repair capacity of bone erosion, the inhibition of osteoblasts differentiation and function may cause to impaired bone formation in RA. There are two main pathways required for osteoblasts differentiation from mesenchymal precursors: Wnt and bone morphogenetic protein (BMP) pathway (Gravallese, 2017). In addition, systemic hormones, fibroblast growth factor, as well as other signaling factors are also involved (Valenti et al., 2018). Several evidences support that Wnt signaling plays a role in bone loss and new bone formation in RA (Cici et al., 2019). In the absence of Wnt stimulation, β-catenin is phosphorylated by the glycogen synthase kinase 3β (GSK-3β) complex and further degraded in the cytoplasm after ubiquitination. While Wnt proteins stimulation inhibit GSK-3β, induce β-catenin accumulation in the cytoplasm, promote β-catenin to enter the nucleus and induce target gene expression (Maeda et al., 2019). Wnt pathway can activate target genes, promote differentiation in pre-osteoblasts, up-regulate OPG expression and down-regulate RANKL expression in osteoblasts (Cici et al., 2019). Wnt signaling is regulated by different inhibitors, such as the secreted frizzled-related protein, the Wnt inhibitory factor 1, sclerostin and the Dickkopf (Dkk) family of secreted proteins. Bone destruction caused by RA may be related to over-production of Wnt inhibitors such as Dkk-1 (Adami et al., 2016). Bone morphogenetic proteins (BMPs) belong to the transforming growth factor-β (TGF-β) super-family. BMP pathway, including canonical drosophila mothers against decapentaplegic protein (Smad) signaling and non-Smad signaling (Sanchez-Duffhues et al., 2015), regulates the proliferation, differentiation, maturation and activity of osteoblasts in bone formation. Inflammatory cytokines in inflamed joints can regulate BMP signaling, and altered BMP signaling is essential for osteoblasts and their progenitors (Lukac et al., 2020). Therefore, targeting the BMP pathway may become a potential treatment for bone destruction in RA (He X. et al., 2019).

## Effects of MiRNAs on Bone Destruction in RA

### MiRNAs Regulate Osteoclasts

#### RANK/RANKL/OPG Signal Pathway

RANK/RANKL/OPG signal pathway plays an important role in osteoclast differentiation. One study showed that the expression level of miR-145-5p in peripheral blood mononuclear cells and synovial tissue of patients with RA increased. During the differentiation of mouse monocyte/macrophage RAW264.7 cells, overexpressed miR-145-5p directly targeted OPG and promoted osteoclast differentiation. MiR-145-5p significantly reduced the expression of OPG, while inhibiting miR-145-5p had the opposite effect. Overexpression of miR-145-5p also up-regulated the expression of RANK and RANKL. In the collagen-induced arthritis (CIA) mice, 4 weeks after intravenous injection of mir-145-5p agomir, the bone erosion of ankle joint became worse (Chen Y. et al., 2018). Changes in miR-124 have been found in many inflammatory and immune diseases (Qin et al., 2016). The expression of miR-124 is reduced in various human tumors and has a tumor suppressing effect (Jia et al., 2019; Moghadasi et al., 2020). Ohnuma et al.’s (2019) research found that overexpression of miR-124 inhibited osteoclast differentiation and bone resorption by inhibiting the level of NFATc1 in mouse bone marrow-derived macrophages (BMMs) induced by tumor necrosis factor-α (TNF-α), interleukin 6 (IL-6), OPG and M-CSF. Besides, Nakamachi et al. (2016) discovered that the expression of miR-124 was significantly down-regulated in the joints of adjuvant-induced arthritis (AIA) rats (Nakamachi et al., 2016). Treated with precursor-miR-124 significantly alleviated bone destruction, and also reduced the expression of RANKL, NFATc1 and integrin β1 in AIA rats. *In vitro*, both rat NFATc1 and human NFATc1 were the direct targets of miR-124 to inhibit osteoclast differentiation (Nakamachi et al., 2016). So far, several other studies had shown that miR-146a inhibited osteoclast differentiation in CIA mice (Ammari et al., 2018), which may be related to the expression of proteins related to the RANK/RANKL/OPG signal pathway. Tao et al. (2017) found that the expression of endogenous miR-106b increased in inflammatory mouse joints. Inhibition of miR-106b significantly alleviated the development of arthritis in CIA mice, decreased RANKL:OPG ratio as well as the number and formation of osteoclasts (Tao et al., 2017).

In addition, many other studies showed that miR-338-3p (Niu et al., 2019) and miR-340 (Zhao et al., 2017a) inhibited the differentiation of osteoclasts by targeting the factors related to RANK/RANKL/OPG signaling pathway, while miR-34c (Cong et al., 2017), miR-182 (Miller et al., 2016), and miR-346-3p (Mao et al., 2020) promoted the formation of osteoclasts by participating in the regulation of the expression of genes and proteins related to this pathway. However, these studies were only carried out *in vitro*, lacking the further verification from animal experiments.

Actually, a miRNA could not only regulate the activities of osteoclasts, some other cells that involved in the development of bone destruction in RA, such as FLS, could also influenced by the same miRNA. Wang et al. showed that miR-21 was up-regulated during the osteoclast formation of RAW264.7 cells induced by RANKL, and up-regulation of miR-21 promoted osteoclast differentiation and bone resorption. Overexpression of miR-21 decreased the expression level of PTEN protein and increased the expression of p-AKT and NFATc1 (Wang S. et al., 2020). One study indicated that the level of miR-21 was increased in FLS of CIA rats, and upregulated miR-21 could promote FLS proliferation by facilitating NF-κB nuclear translocation (Chen et al., 2016). However, another research showed that after intraperitoneal injection of miR-21 lentivirus, the paw volume and arthritis index of CIA rats were lower than those of CIA model group. MiR-21 inhibited the expression of IL-6, IL-8, and Wnt protein in synovial tissue of CIA rats. Overexpression of miR-21 alleviated the symptoms of RA by down-regulating Wnt signaling pathway (Liu X. G. et al., 2019). The different conclusions of miR-21 *in vivo* and *in vitro* may be due to the more complex internal environment of RA model, or the differential regulation of miR-21 in different cells. Therefore, these assumptions need to be confirmed by further experiments.

#### M-CSF Related Pathway

M-CSF pathway also plays a key role in the process of osteoclast differentiation. Activated by the combination of M-CSF and M-CSFR, PI3K/AKT pathway can enhance the survival and proliferation of osteoclast precursors (Kim and Kim, 2016). MiR-142 existed in hematopoietic cells and played an important role in inflammation and immune response (Sharma, 2017) and negatively regulated the differentiation and conversion of monocytes and macrophages into osteoclasts (Fordham et al., 2016). Moreover, the expression of miR-142-3p was up-regulated in synovial tissue of RA patients (Qiang et al., 2019). Another study in CIA mice showed that after injection of miR-17-5p mimic, the number of osteoclasts reduced by directly reducing the expression of signal transducer and activator of transcription 3 (STAT3) and janus kinase-1 (JAK1) (Najm et al., 2020). However, the role and function of miRNAs in RA animal models as well as RA patients still need further studies.

#### MiRNAs Regulate Other Factors in Osteoclasts

In addition, as a key cytokine in the pathophysiology of RA, TNF can stimulate osteoclast differentiation (Noack and Miossec, 2017). Anti-TNF drugs may improve the condition of patients with RA (Bek et al., 2017). MiR-125a-5p, known as a tumor suppressor, participates in cancer development and progression by regulating cell proliferation, migration, invasion and metastasis. Studies had shown that the concentration of miR-125a-5p in peripheral blood of patients with RA was obviously increased (Ormseth et al., 2015; Churov et al., 2015). Sun et al. (2019) found that miR-125a-5p increased significantly during the differentiation of RAW 264.7 cells stimulated by M-CSF and RANKL. Overexpression of miR-125a-5p up-regulated the expression levels of translating ribosome affinity purification (TRAP), matrix metalloproteinase-2 (MMP-2), MMP-9 and cathepsin K, inhibited the expression of TNF receptor superfamily member 1B (TNFRSF1B) and promoted the differentiation of osteoclasts (Sun et al., 2019). MiRNA-1225 is regarded as a modulator in the development of cancers and other biological reactions. A research found that the expression of miR−1225 decreased in the differentiation of BMMs stimulated by RANKL. By regulating the axis of Keap1-Nrf2-TNF-α, miR-1225 inhibited the differentiation of osteoclasts, and then regulated the production of reactive oxygen species (Reziwan et al., 2019). However, more *in vivo* experiments still urgently needed to provide further evidences. As anti-TNF drugs have been applied in clinical practice, whether miRNAs-based therapy could act synergistically with anti-TNF therapy on RA may be one of the research trends. In addition, miR-506 could partially target Sirtuin 1 (SIRT1). After inhibiting the expression of miR-506 (Yan et al., 2019), osteoclast formation was inhibited. MiR-199a-5p (Guo K. et al., 2018) promoted osteoclast differentiation by regulating Mafb. Whereas miR-192-5p could inhibit osteoclast formation by negatively regulating the expression of ras-related C3 botulinum toxin substrate 2 (RAC2) in CIA rats (Zheng et al., 2020).

### MiRNAs Regulate Osteoblasts

#### Wnt Pathway

Wnt pathway is one of the important pathways in osteoblast differentiation. β-catenin is the key factor of Wnt pathway, and many miRNAs work directly or indirectly through β-catenin. Lin et al. considered that miR-92a-1-5p was significantly down-regulated during the osteogenic differentiation of mouse osteoblast precursor cell line MC3T3-E1 induced by BMP2. MiR-92a-1-5p was a negative regulator of osteogenic differentiation. The expression of β-catenin was negatively regulated by miR-92a-1-5p, and the inhibition of miR-92a-1-5p on β-catenin was weakened by Wnt signal activator or GSK-3β knockout (Lin Z. et al., 2019). In addition, by regulating the expression of Wnt pathway related factors such as β -catenin, miR-26b-3p (Lin Y. et al., 2019), miR-193a (Wang S. N. et al., 2018; Wang W. et al., 2018), miR-4739 (Elsafadi et al., 2017), miR-150-3p (Wang N. et al., 2016), and miR-23a (Li et al., 2016) could also inhibit osteoblast differentiation but miR-101 (Wang H. et al., 2016) and miR-199b-5p (Zhao et al., 2016) could promote osteoblast differentiation.

Besides regulating β-catenin, some miRNAs could also influence Wnt pathway inhibitors. As a Wnt antagonist, DKK2 inhibits osteoblast activity by binding low-density-lipoprotein receptor-related proteins 5/6 (LRP5/6), thus preventing the formation of Wnt-FZD-LRP complex. Additionally, Myd88 signal can inhibit proliferation, migration and differentiation of BMSCs by inhibiting AKT/β-catenin/GSK-3β signal pathway (Martino et al., 2016). Xia et al. (2019) pointed out that overexpressed miR-200c enhanced the osteoblast differentiation of human BMSCs. Overexpressed miR-200c enhanced the levels of BMP2, Runx2, RANKL, Osterix(Osx), Osteocalcin (OCN), Osteopontin (OPN) and collagen type I by directly decreasing Myd88, as well as the expression of β-catenin, AKT and phosphorylated AKT. After inhibiting miR-200c, the effect was opposite (Xia et al., 2019).

#### BMP Pathway

Another important pathway of osteoblast differentiation is BMP pathway. Members of TGF-β superfamily transmit intracellular signals through Smad complex or MAPK cascade. In Smad-dependent signaling, phosphorylated Smad2/3 or phosphorylated Smad1/5/8 is complexed with Smad4 and co-located in the nucleus, where they regulated the expression of downstream target genes (Wu M. et al., 2016). Chen et al. (2020) pointed out that the differentiation and mineralization of primary osteoblasts isolated from a RA patient were inhibited after co-culture with exosomes of fibroblast-like synoviocytes from synovial tissues. Exosomes treated with miR-486-5p mimic promoted osteoblast differentiation, and increased BMP2 expression and Smad1/5/8 phosphorylation by decreasing Tob1 expression. In CIA mice, the exosomes of synovial fibroblasts also confirmed the same results (Chen et al., 2020). However, the role of miR-486-5p in RA animal models still need further investigation.

There are also miRNAs that affect Samd7 which negatively regulates BMP pathway. He G. et al. (2019) showed that miR-877-3p promoted the osteogenic differentiation and mineralization of MC3T3-E1 cells induced by TGF-β1, and inhibited the expression of Smad7 gene. MiR-877-3p promoted the expression of osteogenic genes such as Runx2, Osx, collagen type I alpha 1, p-Smad2, and p-Smad3, but this promotion was significantly inhibited by Smad7. The expression of Smad7 was up-regulated and the levels of p-Smad2 and p-Smad3 were decreased by inhibiting miR-877-3p (He G. et al., 2019). Additionally, by regulating the translation and expression of BMP pathway related factors, miR-144-3p (Huang C. et al., 2016), miR-217 (Zhu et al., 2017), miR-92a (Yan et al., 2018), and miR-765 (Wang T. et al., 2020) also inhibited osteoblast differentiation.

#### MiRNAs Regulate Other Factors in Osteoblasts

In addition to Wnt and BMP pathways, osteoblast differentiation is also regulated by many other factors. Many miRNAs inhibited the osteogenic differentiation of cells in various ways. For example, miR-383 (Tang et al., 2018) directly regulated Satb2, miR-27a (Gong et al., 2016) reduced the protein and gene expression level of Sp7, miR-10b (Yang et al., 2017) overexpression up-regulated the expression of signal transducer and activator of transcription 1 (STAT1) and blocked the nuclear translocation of Runx2, miR-23a-5p (Ren et al., 2018; Yang et al., 2020) directly targeted and inhibited the expression of Runx2 and MAPK13, miR-206 suppressed the glutamine metabolism (Chen et al., 2019), miR-320a (Huang J. et al., 2016) negatively regulated homeobox a10 (HOXA10), miR-214 (Guo et al., 2017) participates in the inhibition of the c-jun n-terminal kinase (JNK) and p38 pathways, miR-495 (Tian et al., 2017) directly targeted the high-mobility group A2 gene (HMGA2), miR-125a-3p negatively regulated G-protein-coupled receptor kinase interacting protein-1 (GIT1) (Tu et al., 2016), and after miR-223 (Zhang et al., 2018) and miR-23a cluster (Godfrey et al., 2018) inhibition, short-chain dehydrogenase/reductase 3 (DHRS3) and HoxA cluster were respectively targeted to promote osteogenic differentiation.

On the contrary, there are also many other miRNAs that can promote the osteogenic differentiation of cells. MiR-1-3p (Zhou et al., 2020) directly targeted and inhibited the expression of hypoxia-inducible factor 1-alpha inhibitor (HIF1AN), miR-5100 (Wang et al., 2017) directly inhibited the expression of Tob2, miR-7-5p (Chen B. et al., 2018) directly targeted chemokine-like receptor 1 (CMKLR1), miR-590-3p (Wu S. et al., 2016) directly inhibited the expression of adenomatous polyposis coli (APC), and miR-98 promoted osteogenic differentiation via targeting HMGA2 in human BMSCs (Gao et al., 2018).

Liu et al. (2018) Interestingly, in one study, miR-98-5p negatively regulated the expression of casein kinase 2 interacting protein-1 (CKIP-1) gene and protein to promote the osteogenic differentiation of MC3T3-E1 cells (Liu et al., 2018). But another study in three types of cells (MC3T3-E1 cells, mouse and human BMSCs), miR-98-5p inhibited osteogenic differentiation by targeting HMGA2 (Zheng et al., 2019). And for miR-224, it inhibited osteoblast differentiation in mesenchymal stem cells by directly targeting Smad4 (Luo et al., 2018), but miR-224 regulated the expression of Rac1 to promoted osteogenic differentiation in human mesenchymal stem cells (Cai et al., 2019).

### MiRNAs Dual-Regulate Osteoclasts and Osteoblasts

With the deepening of the studies, researchers found that some miRNAs not only act on a single pathway or a kind of cell. They can simultaneously regulate osteoclasts and osteoblasts as well.

Takigawa et al. (2016) held that miR-222-3p, as an inhibitory regulator of osteoclast generation, inhibited the up-regulation of TRAP and cathepsin K in RAW264.7 cells. MiR-222-3p significantly down-regulated the gene level of NFATc1. Yan et al. (2016) manifested that miR-222-3p inhibited the osteogenic differentiation of human BMSCs. Overexpression of miR-222-3p inhibited Smad5 and Runx2 protein levels, while miR-222-3p had the opposite inhibitory effect and stimulated the phosphorylation of Smad1/5/8 (Yan et al., 2016).

Zhao et al. (2017b) discussed that in mouse BMMs cells, TGF-β1 up-regulated the expression of miR-155 and inhibited osteoclast differentiation, while silencing Smad4 reversed the effect of TGF-β1. MiR-155 inhibited osteoclast differentiation by targeting two regulators of osteoclast generation, suppressor of cytokine signaling and microphthalmia-associated transcription factor (Zhao et al., 2017b). MiR-155 not only inhibited osteoclast differentiation, but also inhibited osteoblast differentiation. Gu et al. (2017) showed that in MC3T3-E1 cells induced by BMP2, miR-155 was down-regulated. The gene and protein expression of p-Smad5 and Smad5 was blocked after miR-155 overexpression. Overexpression of Smad5 reversed the inhibitory effect of miR-155 on osteogenesis (Gu et al., 2017).

Lee et al. (2020) indicated that intraarticular injection of miR-9 not only reduced the clinical arthritis score and erosion score, but also significantly decreased inflammatory exudate, inflammation rate and pannus formation, and inhibited osteoclast formation in CIA rats. Qu et al. (2016) thought that miR-9 promoted osteogenic differentiation in MC3T3-E1 cells induced by BMP2. Overexpression of miR-9 up-regulated the concentration of p-adenosine monophosphate-activated protein kinase (p-AMPK) and promoted the protein expression of p-AMPK to increase significantly (Qu et al., 2016).

Therefore, a miRNA may inhibit or promote osteoclast and osteogenesis at the same time, or it may have an opposite effect on osteoclasts and osteoblasts. Maybe the concentration, action time and microenvironment can partly explain the diversity of miRNAs’ action.

## Conclusion and Prospect

In RA, the cure rate of bone destruction is low and the disability rate is high, which seriously threatens the daily life of RA patients. Conventional synthetic disease-modified antirheumatic drugs (csDMARDs), as the first-line drugs recommended by domestic and foreign guidelines, can effectively alleviate the progress of RA and control clinical symptoms, but they are not effective for all RA patients (Schett et al., 2016). The effects of csDMARDs on bone destruction caused by RA are not as expected. The emergence and application of biological agents has brought a major breakthrough in the treatment of bone destruction in RA. Although the use of drugs can alleviate clinical symptoms to a certain extent, it cannot completely repair bone destruction. In the current studies of treatment methods, researchers mainly focus on inflammation control and osteoclast inhibition. However, for the bone destruction caused by RA, this is not enough. The repair of bone destruction and new bone formation may be an important part of the future treatment plan. Additionally, although the ability of miRNAs to target multiple genes in the process of osteoclast and osteoblast differentiation makes them a promising target for developing drugs to treat bone destruction in RA, most of the current researches on miRNAs regulating bone destruction were mainly *in vitro* experiments, lacking enough evidences from animal experiments and clinical trials. Therefore, further animal and clinical researches will be the focus of the next studies. However, the complex role of miRNAs makes further animal experiments and clinical trials face great challenges. As mentioned above, the role of miRNAs may be influenced by different time of action and different concentrations in different microenvironments. Our understanding of miRNAs is still far from enough. How to better and faster make miRNAs-based drugs play a role in the treatment of RA bone destruction is a question we need to carefully think about. Due to the complexity of the role of miRNAs in different pathways, different cells and different microenvironment, some technologies such as omics, systemic biology and biological computing need to be jointly introduced in in-depth studies. In addition, since the ultimate purposes of basic researches are to apply and serve the clinical practices, we should also consider more miRNAs supported by clinical data (Wang M. et al., 2020) in future studies, so as to continuously improve our understanding of miRNAs in bone destruction of RA.

**FIGURE 1:**
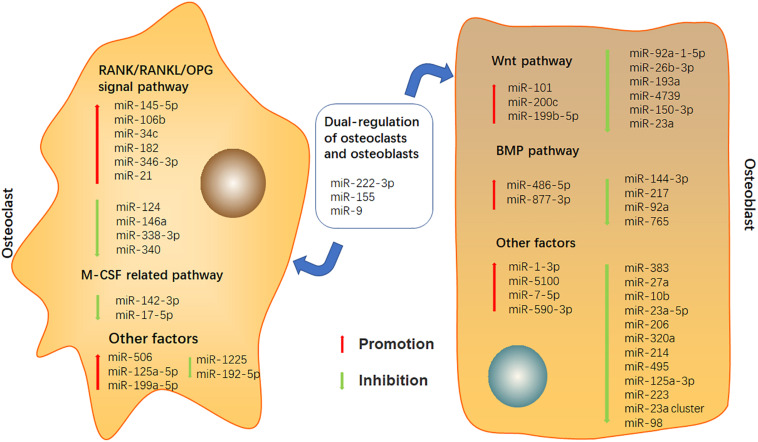
Effects of miRNAs on osteoclast and osteoblast.

In this article, we summarized the potential of miRNAs involved in bone destruction, provided the latest overview of miRNAs in bone metabolism, and demonstrated the role of miRNAs in osteoclast and osteoblast differentiation of RA (Figure 1 and Supplementary Table 1). Although a large number of further studies are still needed to discover the complete miRNAs information network regulating bone balance, targeting miRNAs can be considered as a potential candidate for the treatment of bone destruction caused by RA in future.

## Author Contributions

HZ wrote the manuscript. AL and XH revised and approved the manuscript. All authors contributed to the article and approved the submitted version.

## Conflict of Interest

The authors declare that the research was conducted in the absence of any commercial or financial relationships that could be construed as a potential conflict of interest.
