# Supplementary material for: Roles of MicroRNAs in Bone Destruction of Rheumatoid Arthritis
Source: Front Cell Dev Biol. 2020 Nov 19;8:600867. doi: 10.3389/fcell.2020.600867 (PMC7710907; doi:10.3389/fcell.2020.600867)
Supplement: Supplementary file 1 [file Table_1.DOCX]

**Supplementary Table 1 Roles of MicroRNAs in Bone Destruction**

| **miRNAs** | **Model** | **Targets** | **Results** | **References** |
| --- | --- | --- | --- | --- |
| miR-145-5p | RAW-264.7 cells; CIA mice | OPG | promoted osteoclastic differentiation of RAW264.7 cells; aggravated bone erosion in CIA mice | (Chen,Wang, et al. 2018) |
| miR-106b | CIA mice | - | miR-106b inhibition can ameliorate bone destruction | (Tao et al. 2017) |
| miR-34c | mouse BMMs and RAW 264.7 cells | LGR4 | promoted osteoclast differentiation | (Cong et al. 2017) |
| miR-182 | mouse BMMs | Foxo3, Maml1 | enhanced osteoclastogenesis | (Miller et al. 2016) |
| miR-346-3p | RAW 264.7 cells | TRAF3 | promoted osteoclastogenesis | (Mao et al. 2020) |
| miR-21 | RAW264.7 cells | PTEN | promoted osteoclastogenesis and bone resorption | (Wang,Liu, et al. 2020) |
| miR-124 | mouse BMMs; AIA rats | NFATc1 | inhibited osteoclastogenesis | (Ohnuma et al. 2019; Nakamachi et al. 2016) |
| miR-146a | CIA mice | Relb | attenuated bone erosion in arthritis | (Ammari et al. 2018) |
| miR-338-3p | RAW 264.7 cells | IKKβ | inhibited the formation and absorption activity of osteoclasts | (Niu et al. 2019) |
| miR-340 | mouse BMMs | MITF | inhibited osteoclast differentiation | (Zhao et al. 2017a) |
| miR-142-3p | human monocytes and macrophages | - | inhibited osteoclast differentiation | (Fordham et al. 2016) |
| miR-17-5p | CIA mice | STAT3, JAK1 | reduced in the number of osteoclasts | (Najm et al. 2020) |
| miR-506 | mouse BMMs | SIRT1 | osteoclast formation was inhibited after inhibition of miR-506 expression | (Yan et al. 2019) |
| miR-125a-5p | RAW 264.7 cells | TNFRSF1B | promoted the differentiation of osteoclasts | (Sun et al. 2019) |
| miR‐199a‐5p | mouse BMMs and RAW 264.7 cells | Mafb | promoted osteoclast differentiation | (Guo et al. 2018) |
| miR-1225 | mouse BMMs | Keap1 | inhibited osteoclastogenesis | (Reziwan et al. 2019) |
| miR-192-5p | CIA rats | RAC2 | inhibited osteoclast formation | (Zheng et al. 2020) |
| miR-101 | human BMSCs | EZH2 | promoted the osteoblast differentiation | (Wang,Meng, et al. 2016) |
| miR‐200c | human BMSCs | Myd88 | promoted osteogenic differentiation | (Xia et al. 2019) |
| miR-199b-5p | human BMSCs | GSK-3β | promoted osteogenic differentiation | (Zhao et al. 2016) |
| miR-92a-1-5p | MC3T3-E1 cells | β‑catenin | inhibited osteogenic differentiation | (Lin,Tang, et al. 2019) |
| miR-26b-3p | MC3T3-E1 cells | ER-α | suppressed osteoblast differentiation | (Lin,Xiao, et al. 2019) |
| miR-193a | mouse BMSCs;  human BMSCs | LGR4, HMGB1 | impeded osteoblast differentiation | (Wang,Chen, et al. 2018; Wang,Zhao, et al. 2018) |
| miR-4739 | human BMSCs | LRP3 | suppressed osteogenic differentiation | (Elsafadi et al. 2017) |
| miR-150-3p | human BMSCs | β-catenin | inhibited osteogenic differentiation | (Wang,Zhou, et al. 2016) |
| miR-23a | human BMSCs | LRP5 | inhibited osteoblast differentiation | (Li et al. 2016) |
| miR-486-5p | human primary osteoblasts | Tob1 | promoted osteoblast differentiation | (Chen et al. 2020) |
| miR-877-3p | MC3T3-E1 cells | Smad7 | promoted the osteoblastic differentiation | (He et al. 2019) |
| miR-144-3p | C3H10T1/2 cells | Smad4 | inhibited osteogenic differentiation | (Huang,Geng, et al. 2016) |
| miR‑217 | rat BMSCs | Runx2 | inhibited osteogenic differentiation | (Zhu et al. 2017) |
| miR-92a | mouse BMSCs | Smad6 | inhibited the osteogenic differentiation | (Yan et al. 2018) |
| miR-765 | human BMSCs | BMP6 | inhibited osteogenic differentiation | (Wang,Zhang, et al. 2020) |
| miR-1-3p | MC3T3-E1 cells | HIF1AN | promoted osteogenic differentiation | (Zhou et al. 2020) |
| miR-5100 | ST2 and MC3T3-E1 cells | Tob2 | promoted osteogenic differentiation | (Wang et al. 2017) |
| miR-7-5p | human BMSCs | CMKLR1 | promoted osteogenic differentiation | (Chen,Meng, et al. 2018) |
| miR-590-3p | human mesenchymal stem cells | APC | promoted osteogenic differentiation | (Wu et al. 2016) |
| miR-383 | rat BMSCs | Satb2 | suppressed osteoblastic differentiation | (Tang et al. 2018) |
| miR-27a | mouse BMSCs | Sp7 | inhibited osteogenic differentiation | (Gong et al. 2016) |
| miR-10b | MC3T3-E1 cells | Bcl6 | inhibited osteoblast differentiation | (Yang et al. 2017) |
| miR-23a-5p | MC3T3-E1 cells; hFOB1.19 cells | Runx2 | inhibited osteoblast activity | (Yang et al. 2020) |
|  | human BMSCs | MAPK13 | inhibited osteoblast differentiation | (Ren et al. 2018) |
| miR-206 | human BMSCs | glutaminase | inhibited osteoblast differentiation | (Chen et al. 2019) |
| miR-320a | human BMSCs | HOXA10 | inhibited osteogenesis | (Huang,Meng, et al. 2016) |
| miR-214 | rat BMSCs | - | inhibited the osteogenic differentiation | (Guo et al. 2017) |
| miR-495 | murine calvarial osteoblasts | HMGA2 | inhibited osteogenic differentiation | (Tian et al. 2017) |
| miR-125a-3p | human BMSCs | GIT1 | inhibited osteoblastic proliferation and differentiation | (Tu et al. 2016) |
| miR-223 | human BMSCs | DHRS3 | inhibition of miR-223 promoted the osteogenic differentiation | (Zhang et al. 2018) |
| miR-23a cluster | MC3T3-E1 cells; miR-23a cluster knockdown female mice | HoxA cluster | inhibition of the miR-23a cluster promoted osteoblast differentiation | (Godfrey et al. 2018) |
| miR-98 | human BMSCs | HMGA2 | promoted osteogenic differentiation | (Gao et al. 2018) |
| miR-98-5p | MC3T3-E1 cells | CKIP-1 | promoted osteoblast differentiation | (Liu et al. 2018) |
|  | MC3T3-E1 cells, mouse and human BMSCs | HMGA2 | inhibited osteogenic differentiation | (Zheng et al. 2019) |
| miR-224 | mesenchymal stem cells | Smad4 | inhibited osteoblast differentiation | (Luo et al. 2018) |
|  | human mesenchymal stem cells | Rac1 | promoted osteogenic differentiation | (Cai et al. 2019) |
| miR-222-3p | RAW264.7 cells | NFATc1 | inhibited osteoclast generation | (Takigawa et al. 2016) |
|  | human BMSCs | Smad5, RUNX2 | inhibited osteogenic differentiation | (Yan et al. 2016) |
| miR-155 | mouse BMMs | SOCS1, MITF | inhibited osteoclast differentiation | (Zhao et al. 2017b) |
|  | MC3T3-E1 cells | Smad5 | inhibited osteoblast differentiation | (Gu et al. 2017) |
| miR-9 | CIA rats | NF-κB1 | inhibited osteoclast formation | (Lee et al. 2020) |
|  | MC3T3-E1 cells | pAMPK | promoted osteogenic differentiation | (Qu et al. 2016) |

**References:**

Ammari, M., Presumey, J., Ponsolles, C., Roussignol, G., Roubert, C., Escriou, V., et al.(2018).Delivery of miR-146a to Ly6C(high) Monocytes Inhibits Pathogenic Bone Erosion in Inflammatory Arthritis. Theranostics 8**,** 5972-85.doi:10.7150/thno.29313

Cai, Q., Zheng, P., Ma, F., Zhang, H., Li, Z., Fu, Q., et al.(2019).MicroRNA-224 enhances the osteoblastic differentiation of hMSCs via Rac1. Cell Biochem Funct 37**,** 62-71.doi:10.1002/cbf.3373

Chen, B., Meng, J., Zeng, Y. T., Du, Y. X., Zhang, J., Si, Y. M., et al.(2018).MicroRNA-7-5p regulates osteogenic differentiation of hMSCs via targeting CMKLR1. Eur Rev Med Pharmacol Sci 22**,** 7826-31.doi:10.26355/eurrev_201811_16407

Chen, J., Liu, M., Luo, X., Peng, L., Zhao, Z., He, C., et al.(2020).Exosomal miRNA-486-5p derived from rheumatoid arthritis fibroblast-like synoviocytes induces osteoblast differentiation through the Tob1/BMP/Smad pathway. Biomater Sci 8**,** 3430-42.doi:10.1039/c9bm01761e

Chen, Y., Wang, X., Yang, M., Ruan, W., Wei, W., Gu, D., et al.(2018).miR-145-5p Increases Osteoclast Numbers In Vitro and Aggravates Bone Erosion in Collagen-Induced Arthritis by Targeting Osteoprotegerin. Med Sci Monit 24**,** 5292-300.doi:10.12659/MSM.908219

Chen, Y., Yang, Y. R., Fan, X. L., Lin, P., Yang, H., Chen, X. Z., et al.(2019).miR-206 inhibits osteogenic differentiation of bone marrow mesenchymal stem cells by targetting glutaminase. Biosci Rep 39.doi:10.1042/bsr20181108

Cong, F., Wu, N., Tian, X., Fan, J., Liu, J., Song, T., et al.(2017).MicroRNA-34c promotes osteoclast differentiation through targeting LGR4. Gene 610**,** 1-8.doi:10.1016/j.gene.2017.01.028

Elsafadi, M., Manikandan, M., Alajez, N. M., Hamam, R., Dawud, R. A., Aldahmash, A., et al.(2017).MicroRNA-4739 regulates osteogenic and adipocytic differentiation of immortalized human bone marrow stromal cells via targeting LRP3. Stem Cell Res 20**,** 94-104.doi:10.1016/j.scr.2017.03.001

Fordham, J. B., Guilfoyle, K., Naqvi, A. R., and Nares, S.(2016).MiR-142-3p is a RANKL-dependent inducer of cell death in osteoclasts. Sci Rep 6**,** 24980.doi:10.1038/srep24980

Gao, X. L., Cao, M. G., Ai, G. G., and Hu, Y. B.(2018).Mir-98 reduces the expression of HMGA2 and promotes osteogenic differentiation of mesenchymal stem cells. Eur Rev Med Pharmacol Sci 22**,** 3311-17.doi:10.26355/eurrev_201806_15150

Godfrey, T. C., Wildman, B. J., Beloti, M. M., Kemper, A. G., Ferraz, E. P., Roy, B., et al.(2018).The microRNA-23a cluster regulates the developmental HoxA cluster function during osteoblast differentiation. J Biol Chem 293**,** 17646-60.doi:10.1074/jbc.RA118.003052

Gong, Y., Lu, J., Yu, X., and Yu, Y.(2016).Expression of Sp7 in Satb2-induced osteogenic differentiation of mouse bone marrow stromal cells is regulated by microRNA-27a. Mol Cell Biochem 417**,** 7-16.doi:10.1007/s11010-016-2709-y

Gu, Y., Ma, L., Song, L., Li, X., Chen, D., and Bai, X.(2017).miR-155 Inhibits Mouse Osteoblast Differentiation by Suppressing SMAD5 Expression. Biomed Res Int 2017**,** 1893520.doi:10.1155/2017/1893520

Guo, K., Zhang, D., Wu, H., Zhu, Q., Yang, C., and Zhu, J.(2018).MiRNA-199a-5p positively regulated RANKL-induced osteoclast differentiation by target Mafb protein. J Cell Biochem.doi:10.1002/jcb.27968

Guo, Y., Li, L., Gao, J., Chen, X., and Sang, Q.(2017).miR-214 suppresses the osteogenic differentiation of bone marrow-derived mesenchymal stem cells and these effects are mediated through the inhibition of the JNK and p38 pathways. Int J Mol Med 39**,** 71-80.doi:10.3892/ijmm.2016.2826

He, G., Chen, J., and Huang, D.(2019).miR-877-3p promotes TGF-β1-induced osteoblast differentiation of MC3T3-E1 cells by targeting Smad7. Exp Ther Med 18**,** 312-19.doi:10.3892/etm.2019.7570

Huang, C., Geng, J., Wei, X., Zhang, R., and Jiang, S.(2016).MiR-144-3p regulates osteogenic differentiation and proliferation of murine mesenchymal stem cells by specifically targeting Smad4. FEBS Lett 590**,** 795-807.doi:10.1002/1873-3468.12112

Huang, J., Meng, Y., Liu, Y., Chen, Y., Yang, H., Chen, D., et al.(2016).MicroRNA-320a Regulates the Osteogenic Differentiation of Human Bone Marrow-Derived Mesenchymal Stem Cells by Targeting HOXA10. Cell Physiol Biochem 38**,** 40-8.doi:10.1159/000438607

Lee, Wen Shi, Yasuda, Shinsuke, Kono, Michihiro, Kudo, Yuki, Shimamura, Sanae, Kono, Michihito, et al.(2020).MicroRNA-9 ameliorates destructive arthritis through down-regulation of NF-κB1-RANKL pathway in fibroblast-like synoviocytes. Clinical Immunology 212.doi:10.1016/j.clim.2020.108348

Li, T., Li, H., Wang, Y., Li, T., Fan, J., Xiao, K., et al.(2016).microRNA-23a inhibits osteogenic differentiation of human bone marrow-derived mesenchymal stem cells by targeting LRP5. Int J Biochem Cell Biol 72**,** 55-62.doi:10.1016/j.biocel.2016.01.004

Lin, Y., Xiao, L., Zhang, Y., Li, P., Wu, Y., and Lin, Y.(2019).MiR-26b-3p regulates osteoblast differentiation via targeting estrogen receptor α. Genomics 111**,** 1089-96.doi:10.1016/j.ygeno.2018.07.003

Lin, Z., Tang, Y., Tan, H., and Cai, D.(2019).MicroRNA-92a-1-5p influences osteogenic differentiation of MC3T3-E1 cells by regulating β-catenin. J Bone Miner Metab 37**,** 264-72.doi:10.1007/s00774-018-0935-y

Liu, Q., Guo, Y., Wang, Y., Zou, X., and Yan, Z.(2018).miR‑98‑5p promotes osteoblast differentiation in MC3T3‑E1 cells by targeting CKIP‑1. Mol Med Rep 17**,** 4797-802.doi:10.3892/mmr.2018.8416

Luo, Y., Cao, X., Chen, J., Gu, J., Zhao, J., and Sun, J.(2018).MicroRNA-224 suppresses osteoblast differentiation by inhibiting SMAD4. J Cell Physiol 233**,** 6929-37.doi:10.1002/jcp.26596

Mao, Y., Chen, Y., Fu, Y., Guan, J., Liang, M., Zhu, Y., et al.(2020).miR-346-3p promotes osteoclastogenesis via inhibiting TRAF3 gene. In Vitro Cell Dev Biol Anim 56**,** 533-42.doi:10.1007/s11626-020-00479-w

Miller, C. H., Smith, S. M., Elguindy, M., Zhang, T., Xiang, J. Z., Hu, X., et al.(2016).RBP-J-Regulated miR-182 Promotes TNF-alpha-Induced Osteoclastogenesis. J Immunol 196**,** 4977-86.doi:10.4049/jimmunol.1502044

Najm, A., Masson, F. M., Preuss, P., Georges, S., Ory, B., Quillard, T., et al.(2020).miR-17-5p reduces inflammation and bone erosions in collagen induced arthritis mice and directly targets the JAK-STAT pathway in rheumatoid arthritis fibroblast-like synoviocytes. Arthritis Rheumatol.doi:10.1002/art.41441

Nakamachi, Y., Ohnuma, K., Uto, K., Noguchi, Y., Saegusa, J., and Kawano, S.(2016).MicroRNA-124 inhibits the progression of adjuvant-induced arthritis in rats. Ann Rheum Dis 75**,** 601-8.doi:10.1136/annrheumdis-2014-206417

Niu, D., Gong, Z., Sun, X., Yuan, J., Zheng, T., Wang, X., et al.(2019).miR-338-3p regulates osteoclastogenesis via targeting IKKβ gene. In Vitro Cell Dev Biol Anim 55**,** 243-51.doi:10.1007/s11626-019-00325-8

Ohnuma, K., Kasagi, S., Uto, K., Noguchi, Y., Nakamachi, Y., Saegusa, J., et al.(2019).MicroRNA-124 inhibits TNF-alpha- and IL-6-induced osteoclastogenesis. Rheumatol Int 39**,** 689-95.doi:10.1007/s00296-018-4218-7

Qu, J., Lu, D., Guo, H., Miao, W., Wu, G., and Zhou, M.(2016).MicroRNA-9 regulates osteoblast differentiation and angiogenesis via the AMPK signaling pathway. Mol Cell Biochem 411**,** 23-33.doi:10.1007/s11010-015-2565-1

Ren, G., Sun, J., Li, M. M., Zhang, Y. D., Li, R. H., and Li, Y. M.(2018).MicroRNA-23a-5p regulates osteogenic differentiation of human bone marrow-derived mesenchymal stem cells by targeting mitogen-activated protein kinase-13. Mol Med Rep 17**,** 4554-60.doi:10.3892/mmr.2018.8452

Reziwan, K., Sun, D., Zhang, B., and Zhao, Z.(2019).MicroRNA-1225 activates Keap1-Nrf2-HO-1 signalling to inhibit TNFα-induced osteoclastogenesis by mediating ROS generation. Cell Biochem Funct 37**,** 256-65.doi:10.1002/cbf.3394

Sun, L., Lian, J. X., and Meng, S.(2019).MiR-125a-5p promotes osteoclastogenesis by targeting TNFRSF1B. Cell Mol Biol Lett 24**,** 23.doi:10.1186/s11658-019-0146-0

Takigawa, S., Chen, A., Wan, Q., Na, S., Sudo, A., Yokota, H., et al.(2016).Role of miR-222-3p in c-Src-Mediated Regulation of Osteoclastogenesis. International journal of molecular sciences 17**,** 240.doi:10.3390/ijms17020240

Tang, J., Zhang, Z., Jin, X., and Shi, H.(2018).miR-383 negatively regulates osteoblastic differentiation of bone marrow mesenchymal stem cells in rats by targeting Satb2. Bone 114**,** 137-43.doi:10.1016/j.bone.2018.06.010

Tao, Y., Wang, Z., Wang, L., Shi, J., Guo, X., Zhou, W., et al.(2017).Downregulation of miR-106b attenuates inflammatory responses and joint damage in collagen-induced arthritis. Rheumatology (Oxford) 56**,** 1804-13.doi:10.1093/rheumatology/kex233

Tian, Z., Zhou, H., Xu, Y., and Bai, J.(2017).MicroRNA-495 Inhibits New Bone Regeneration via Targeting High Mobility Group AT-Hook 2 (HMGA2). Med Sci Monit 23**,** 4689-98.doi:10.12659/msm.904404

Tu, X. M., Gu, Y. L., and Ren, G. Q.(2016).miR-125a-3p targetedly regulates GIT1 expression to inhibit osteoblastic proliferation and differentiation. Exp Ther Med 12**,** 4099-106.doi:10.3892/etm.2016.3874

Wang, H., Cui, Y., Luan, J., Zhou, X., Li, C., Li, H., et al.(2017).MiR-5100 promotes osteogenic differentiation by targeting Tob2. J Bone Miner Metab 35**,** 608-15.doi:10.1007/s00774-016-0799-y

Wang, H., Meng, Y., Cui, Q., Qin, F., Yang, H., Chen, Y., et al.(2016).MiR-101 Targets the EZH2/Wnt/β-Catenin the Pathway to Promote the Osteogenic Differentiation of Human Bone Marrow-Derived Mesenchymal Stem Cells. Sci Rep 6**,** 36988.doi:10.1038/srep36988

Wang, N., Zhou, Z., Wu, T., Liu, W., Yin, P., Pan, C., et al.(2016).TNF-α-induced NF-κB activation upregulates microRNA-150-3p and inhibits osteogenesis of mesenchymal stem cells by targeting β-catenin. Open Biol 6.doi:10.1098/rsob.150258

Wang, S., Liu, Z., Wang, J., Ji, X., Yao, Z., and Wang, X.(2020).miR21 promotes osteoclastogenesis through activation of PI3K/Akt signaling by targeting Pten in RAW264.7 cells. Mol Med Rep 21**,** 1125-32.doi:10.3892/mmr.2020.10938

Wang, S. N., Zhao, X. Q., Yu, B., and Wang, B. W.(2018).miR-193a inhibits osteogenic differentiation of bone marrow-derived stroma cell via targeting HMGB1. Biochem Biophys Res Commun 503**,** 536-43.doi:10.1016/j.bbrc.2018.05.132

Wang, T., Zhang, C., Wu, C., Liu, J., Yu, H., Zhou, X., et al.(2020).miR-765 inhibits the osteogenic differentiation of human bone marrow mesenchymal stem cells by targeting BMP6 via regulating the BMP6/Smad1/5/9 signaling pathway. Stem Cell Res Ther 11**,** 62.doi:10.1186/s13287-020-1579-0

Wang, W., Chen, J., Hui, Y., Huang, M., and Yuan, P.(2018).Down-regulation of miR-193a-3p promotes osteoblast differentiation through up-regulation of LGR4/ATF4 signaling. Biochem Biophys Res Commun 503**,** 2186-93.doi:10.1016/j.bbrc.2018.08.011

Wu, S., Liu, W., and Zhou, L.(2016).MiR-590-3p regulates osteogenic differentiation of human mesenchymal stem cells by regulating APC gene. Biochem Biophys Res Commun 478**,** 1582-7.doi:10.1016/j.bbrc.2016.08.160

Xia, P., Gu, R., Zhang, W., Shao, L., Li, F., Wu, C., et al.(2019).MicroRNA-200c promotes osteogenic differentiation of human bone mesenchymal stem cells through activating the AKT/β-Catenin signaling pathway via downregulating Myd88. J Cell Physiol 234**,** 22675-86.doi:10.1002/jcp.28834

Yan, J., Guo, D., Yang, S., Sun, H., Wu, B., and Zhou, D.(2016).Inhibition of miR-222-3p activity promoted osteogenic differentiation of hBMSCs by regulating Smad5-RUNX2 signal axis. Biochem Biophys Res Commun 470**,** 498-503.doi:10.1016/j.bbrc.2016.01.133

Yan, S., Miao, L., Lu, Y., and Wang, L.(2019).MicroRNA-506 upregulation contributes to sirtuin 1 inhibition of osteoclastogenesis in bone marrow stromal cells induced by TNF-α treatment. Cell Biochem Funct 37**,** 598-607.doi:10.1002/cbf.3436

Yan, X., Wang, H., Li, Y., Jiang, Y., Shao, Q., and Xu, W.(2018).MicroRNA‑92a overexpression promotes the osteogenic differentiation of bone mesenchymal stem cells by impeding Smad6‑mediated runt‑related transcription factor 2 degradation. Mol Med Rep 17**,** 7821-26.doi:10.3892/mmr.2018.8829

Yang, J., Wang, S., Wang, F., Mu, X., Qu, Y., Zhao, Z., et al.(2017).Downregulation of miR-10b promotes osteoblast differentiation through targeting Bcl6. Int J Mol Med 39**,** 1605-12.doi:10.3892/ijmm.2017.2955

Yang, J. X., Xie, P., Li, Y. S., Wen, T., and Yang, X. C.(2020).Osteoclast-derived miR-23a-5p-containing exosomes inhibit osteogenic differentiation by regulating Runx2. Cell Signal 70**,** 109504.doi:10.1016/j.cellsig.2019.109504

Zhang, S., Liu, Y., Zheng, Z., Zeng, X., Liu, D., Wang, C., et al.(2018).MicroRNA-223 Suppresses Osteoblast Differentiation by Inhibiting DHRS3. Cell Physiol Biochem 47**,** 667-79.doi:10.1159/000490021

Zhao, H., Zhang, J., Shao, H., Liu, J., Jin, M., Chen, J., et al.(2017a).miRNA-340 inhibits osteoclast differentiation via repression of MITF. Biosci Rep 37.doi:10.1042/bsr20170302

Zhao, H., Zhang, J., Shao, H., Liu, J., Jin, M., Chen, J., et al.(2017b).Transforming Growth Factor β1/Smad4 Signaling Affects Osteoclast Differentiation via Regulation of miR-155 Expression. Mol Cells 40**,** 211-21.doi:10.14348/molcells.2017.2303

Zhao, R., Li, Y., Lin, Z., Wan, J., Xu, C., Zeng, Y., et al.(2016).miR-199b-5p modulates BMSC osteogenesis via suppressing GSK-3β/β-catenin signaling pathway. Biochem Biophys Res Commun 477**,** 749-54.doi:10.1016/j.bbrc.2016.06.130

Zheng, F., Wang, F., and Xu, Z.(2019).MicroRNA-98-5p prevents bone regeneration by targeting high mobility group AT-Hook 2. Exp Ther Med 18**,** 2660-66.doi:10.3892/etm.2019.7835

Zheng, J., Zhu, L., Iok In, I., Chen, Y., Jia, N., and Zhu, W.(2020).Bone marrow-derived mesenchymal stem cells-secreted exosomal microRNA-192-5p delays inflammatory response in rheumatoid arthritis. Int Immunopharmacol 78**,** 105985.doi:10.1016/j.intimp.2019.105985

Zhou, L., Qiu, M., Yang, L., Yang, L., Zhang, Y., Mu, S., et al.(2020).MicroRNA-1-3p enhances osteoblast differentiation of MC3T3-E1 cells by interacting with hypoxia-inducible factor 1 α inhibitor (HIF1AN). Mech Dev 162**,** 103613.doi:10.1016/j.mod.2020.103613

Zhu, Y. L., Wang, S., Ding, D. G., Xu, L., and Zhu, H. T.(2017).miR‑217 inhibits osteogenic differentiation of rat bone marrow‑derived mesenchymal stem cells by binding to Runx2. Mol Med Rep 15**,** 3271-77.doi:10.3892/mmr.2017.6349
